# An Appraisal of Methods for Measuring Welfare of Grazing Ruminants

**DOI:** 10.3389/fvets.2019.00289

**Published:** 2019-08-28

**Authors:** Graham K. Barrell

**Affiliations:** Department of Agricultural Sciences, Lincoln University, Christchurch, New Zealand

**Keywords:** animal welfare, ruminants, HPA axis, assessment of pain, animal behavior

## Abstract

Although disturbances in body function of animals can be measured to determine whether a state of stress may exist, there is growing interest in finding ways to assess their emotional status as an indicator of good or bad welfare status. Generally it is easier to determine poor states of well-being than positive ones. For grazing ruminants some indicators of well-being include absence of illness, good growth and productivity, and longevity. Motion detectors can provide automated remote monitoring of behavior and it is likely that there will be advances in the interpretation software to increase the utility of this technology for assessing well-being. Cortisol levels in body fluids, feces and pelage are prominent as a marker of poor animal welfare, but like many of the other objective measures that are used, are not wholly reliable at the individual animal level. These other measures include: plasma serotonin, heart rate variation, infra-red thermography, cytokines, salivary alpha amylase, and acute phase proteins. Use of automated facial expression recognition may supplement electrophysiological recording as means to quantify the pain experience of animals. Although the measures described in the literature do not necessarily provide the final answer for determination of welfare in grazing ruminants, they all have some merit and deserve further investigation.

## Introduction

One of the initial tasks that arises from attempts to quantify the well-being of grazing animals is that of deciding what their well-being, or welfare, actually is. Although this issue is dealt with in some depth in nearby papers, it needs to be addressed here as well to provide context for the measures under consideration. Confusion stems from differing views about what constitutes animal welfare. For instance Moberg, (1) described disturbances of stress in animals as the development of a pre-pathological state, specifically as “a stress-related change in biological function that threatens the animal's well-being,” this being the onset of poor welfare status of an animal. Broom and Johnson (2) provided a wider view that all biological responses represent states of welfare, very good and poor, with the development of pathology as a manifestation of excessive stress and, thus, a poor welfare status. The latter authors (2, 3) also pointed out that most of the quantifiable measures of animals that were being used principally determined poor welfare. What is really needed is a clearer view of the mental state of animals. It is argued that welfare is fulfilled when animals experience positive emotions and do not feel prolonged negative emotions (4–6). This has led more recently to consideration of “animal happiness” where emphasis has shifted from concern about negative aspects of animal welfare to the positives (7). However, the problem remains about how we can interpret the responses of animals in terms of their emotional experiences. It has been addressed by Safina (8) but further discussion of this question and of these definitions lies outside the scope of this paper, which is primarily confined to an examination of the methodology used to evaluate measures of welfare.

The focus of concerns about the welfare of grazing ruminants must center on farmed animals. The great bulk of wild, free-ranging ruminants are only loosely managed by humans and are generally able to experience their normal behaviors. However, they must encounter all manner of situations where their welfare is poor, for example: from predation, during droughts, from wildfires, during blizzards, presence of uncontrolled infectious diseases, etc. Nevertheless, we tend to view this group of animals as beyond our immediate concern in terms of management of their welfare. Apart from farmed animals (and some held as pets or in zoological parks, circuses, etc.) for whose management we are fully responsible, the other group of grazing ruminants for whom welfare is a major concern are those utilized in research projects or in testing procedures. There is an underlying belief amongst animal rights proponents that scientific research involving use of animals is wrong on philosophical grounds, but this belief is often associated with concerns about welfare of the animals. In recent years, many research projects involving ruminant animals have been conducted just for purposes of evaluating measures of their welfare (see later). It is likely that some of these are motivated by the possible need to set welfare standards for the farming of ruminants, as has been experienced by the poultry and pig farming industries—largely in response to concerns raised by the general public.

### Physical Health and Productivity

One generalized view about the determination of a farm animal's well-being is that it can be obtained from their health status and productivity. This concept has been carefully explored by Professor Marian Dawkins in her book “Animal suffering: the science of animal welfare” (9). She pointed out that although the concept that a physically healthy animal must be in a state of good welfare seems attractive, this measure does not provide any evidence about mental well-being of such animals. Nor does high productivity necessarily mean that the animals are experiencing high levels of welfare. In spite of these caveats, the presence of healthy animals with acceptable growth rates and levels of meat, milk or fiber production, and normal reproductive performance must be considered both as sound indicators of good animal welfare and as ones that are readily measured.

One measure that probably integrates both the health status and productivity of farm animals is longevity. It is likely that shortened longevity represents poorer reproductive performance or health status of animals, possibly associated with selection and management practices aimed at high levels of productivity. This appears to be evident in the dairy industries where there has been a reduction of about 50% in the life expectancy of cows in Denmark between 1960 and 1982 (3) and the current life expectancy for dairy cows in Sweden is only 60.5 months (10). These Scandinavian data are likely to be representative of dairy industries globally. It is generally argued that nutritional strategies which improve longevity of ruminants will impact favorably on their welfare (11). Intuitively, longevity of production animals must be fairly easy to determine. This makes it a simple measure that provides a potentially useful indicator of well-being that should be placed high on the list of measures available for assessing animal welfare on farms. Nevertheless, there is a counter argument. Sometimes longevity of animals may come at cost in terms in terms of their welfare and it would be important to recognize when this is the case.

### The Good Life

Another concept of a good animal welfare scenario is that they “are healthy and have what they want” (12). This statement obliges us to determine animals' wants and presupposes that we can determine positive states of emotion. It moves away from the notion that good welfare is simply the absence of negative experiences and forces us to find ways to demonstrate that animals are enjoying positive emotional experiences. This has proven to be difficult, as most studies in the literature for humans and other mammals have focussed on negative emotions such as fear; the reason being that negative experiences are more intense than positive ones and are therefore easier to measure (13). Their review (13) indicated a need at that time for more research on positive affective states in animals, however it seems that this area of study still continues to lack attention (7). Evidence of pleasure can be based on behaviors such as social interaction, reproductive activity, play, self-grooming, anticipatory hyperactivity, and exploration. In many cases these activities are associated with a reward, such as food [e.g., see (14)], environmental enrichment or shelter from inclement weather, and these rewards can be akin, in physiological terms, to those provided by addictive drugs. As with addictive drugs, the underlying physiology of pleasure experience is manifested in activation of specific neural and neuroendocrine pathways which are quantifiable and likely to provide complementary information to that determined from behavioral studies. Some of these are addressed below.

### Threats to Welfare

In all circumstances grazing ruminants experience a wide realm of threats to their welfare. As well as the obvious threats such as inadequate feed or water, inclement weather associated with inadequate shelter and infectious diseases, grazing ruminants may have to contend with competition for space and feed, gastrointestinal parasitism, unsuitable surfaces, lack of feed diversity or variety, toxic plant compounds, predation, and inadequate care from their human minders. In most cases, these are easily identified and can be quantified and managed. The problem occurs when the threats are either not obvious to the observer or when they are below levels of detection, or not considered important. It is in these cases and where we cannot determine whether a threat exists, e.g., limited feed diversity or negative social interactions amongst individuals, that we are fully dependent on the availability of reliable measures of animal well-being.

### Behavioral Assessment to Determine Welfare of Animals

Welfare of animals can be assessed from ethograms of behaviors. This is where the activities of animals are recorded throughout a monitoring period and the amount of time performing each activity provides a spectrum of behavior that can differentiate individuals who are behaving abnormally or even indicate they are undergoing stress. Alternatively, external indicators of behavior, such as skin lesions to quantify aggression in pigs (15), can provide useful assessments of welfare.

The major limitation to behavioral assessment of animals has been the workload demand on the observers, whether conducting direct observations or interrogating hours of closed circuit television recordings. Automation of behavioral monitoring is a rapidly expanding technology that offers much promise for monitoring the welfare of animals, as well as providing measures of their physical health and reproductive status. Use of accelerometers with automated data capture is providing information about activity of animals from devices placed on a leg (16, 17) and/or on a neck collar (17). However, the reliability of the information as an indicator of pain, inflammation or stress does not appear to be very precise. For instance, use of these devices showed that primiparous cows with clinical metritis spent more time on average lying than their metritis-free counterparts although there was no such effect in multiparous cows (16). Likewise, Williams (17) showed a failure of activity-sensing devices to record all potential oestrous events in dairy cows. In the case of a short-term removal of dietary supplement from dairy cows that was insufficient to cause clinical signs of metabolic disorder, these devices revealed a compensatory increase in mean eating and rumination times (18). It is possible that these changes would be more pronounced when the metabolic disturbance reached a point where welfare is compromised, but the current picture emerging from these studies is that the type of information obtained with the automated devices lacks the degree of resolution that would make it reliable for assessing the welfare status of individual animals. Nevertheless, there is promise of increased sophistication of such devices and of data analysis software in this area. For example, linking these recording devices to a real-time location system has enabled cow behavior to be classified more accurately (19). This is an area of technology that is likely to advance rapidly.

Another promising avenue of study is the analysis of farm animal vocalizations (20). Vocal expression by mammals is linked physiologically to their emotions (21) and it is suggested that vocalizations of cattle can be interpreted to assess how they are coping with their farming environment (22).

An interesting behavioral measure that has been applied to sheep is ear posture. Their ears can be scored as “forward,” “backward,” “asymmetric,” or “passive” (23). These authors and Reefman et al. (24) have reported changes in ear posture according to different emotional states in sheep, which makes this measure attractive because of its ease of detection.

### Qualitative Behavior Assessment and Cognitive Bias

An holistic measure of animal welfare based on behavior can be obtained by applying the process of qualitative behavior assessment [e.g., for sheep-(25)]. This involves an initial group assessment to determine whether animals are: relaxed, dejected, thriving, agitated, responsive, dull, content, anxious, bright, vigorous, distressed, then scoring the predominant behavior. Following this, a follow up examination is conducted to count prevalence of several physical indicators of health and welfare (e.g., coughing, lameness, soiling). Collins et al. (26) found this approach useful for evaluation of sheep transport stressors. However, measures of animal behavior are susceptible to the mood of animals at the time of assessment and this is the basis for consideration of cognitive bias, sometimes termed attention bias, during behavioral evaluation of animal welfare. It is not surprising that an animal experiencing a negative affective state, based on its current emotional experiences (or mood), will display different judgment about a stimulus to that of an animal in a positive affected state. This has been ably reviewed recently by Clegg (27) and several studies have examined this topic in respect of studies with sheep (28–31) and calves (32).

### Cortisol

A major component of the stress response of mammals is activation of the hypothalamus-pituitary-adrenal (HPA) axis which manifests as an elevation of circulating levels of β-endorphin, vasopressin and, particularly, cortisol. The stress-induced elevation of β-endorphin levels in blood is related to the stress modulating activity of this and other endogenous opioids (33) and a similar role is performed by vasopressin, in addition to its direct effects on cardiovascular and kidney function (34). Cortisol is a glucocorticoid with an important role in the mobilization of energy stores during activation of the stress process (35). However, cortisol has almost reached “silver bullet” status as the answer to our need for a simple, quantifiable, measure of lowered welfare status for an individual animal. Its measurement in blood plasma has proved useful as a tool to compare various, potentially noxious, farm procedures such as the various techniques for castration of calves [e.g., (36)] and castration and tail docking of lambs [e.g., (37)]. In spite of its universal appeal as a monitor of negative animal welfare status, caution needs to be applied to conclusions based on measurement of cortisol levels in body fluids (38). One factor is the blood sampling procedure itself. Red deer stags blood sampled by jugular venepuncture during manual restraint had a mean plasma cortisol concentration of 56.5 ng/ml which is in stark contrast with the values obtained with a remote blood sampling backpack whilst the stags were on pasture and undisturbed − 8.4 ng/ml (39). These plasma cortisol values obtained from undisturbed animals are low in comparison with other figures in the literature and indicate that even where blood samples are obtained via an indwelling cannula that has been placed intravenously some days prior, the animals are still susceptible to human presence at the time of sampling. Although, gene transcription and eventual synthesis and secretion of *de novo* hormone product may take several minutes, there are ready releasable sources of cortisol—as seen in blood samples collected at 10 min following administration of adrenocorticotrophic hormone (ACTH) or corticotrophin releasing hormone (CRH) in young (3 weeks) and older (26 weeks) calves (40). This means that the arrival of operators to collect blood, albeit remotely, may be a sufficient stimulus to elevate cortisol in the resultant samples. The same will be true for saliva samples. The study by Van Reenen (40) also revealed a lack of consistency between the responsiveness of cortisol to exogenous CRH or ACTH and behavioral tests, and an age-related increase in responsiveness in the calves. It is thus very unlikely that much emphasis can be placed on a single cortisol measurement in a circulating body fluid as a measure of an animal's state of welfare.

Measurement of glucocorticoid metabolites in the feces of mammals provides a non-invasive approach for determination of recent adrenal cortex activity. The methodology for dairy cows has been nicely validated by Catherine Morrow and her co-authors (41). The lag intervals between elevation of plasma corticosteroids and subsequent elevation of metabolite levels in feces approximated digesta intestinal transit times (41). Although the magnitude of the elevations of the metabolites in feces is much lower than that of the corresponding steroid plasma levels, the method is sensitive enough to detect changes on exposure of cows to a new environment and following their transportation (41, 42). Whilst the data obtained from the numerous published studies have been very encouraging, many authors still consider that it is necessary to use this methodology in concert with other monitoring measures to provide reliable indicators of stress.

It can be argued that the information provided by measurement of cortisol, or its metabolites, in blood, saliva, urine or feces is relevant only to the previous few minutes and up to a few days of retrospective experience of the animal. A longer-term picture of HPA axis activity, for instance a period of chronic stress, may be afforded from measurements of these compounds in the hair or wool of animals (43). However, there are several considerations that must be borne in mind regarding cortisol levels in hair. Firstly, skin (melanocytes) and hair follicle cells contain all elements of the HPA axis including signal molecules (pro-opiomelanocortin, corticotrophin releasing hormone, adrenocorticotrophic hormone) and their receptors, plus the steroid synthesis machinery (44). Thus, there is an HPA axis homolog in skin tissues that can produce corticosteroids independently of the central stress axis. Secondly, incorporation of locally derived corticosteroids and those passively acquired from blood into the growing hair shaft takes place at the follicle bulb (45)—several millimeters below the skin surface (46)—so there is considerable delay before they can be located in shaved hair and this is further complicated by variation, especially seasonal, in hair growth rate and skin blood flow. Also, there is possible “washout” of steroids from hair caused by chemical degradation, grooming, ultraviolet radiation, rainfall, etc., and possible contamination from sweat. However, the ease of collection of hair or wool and the stability of its corticosteroid levels during storage makes this an attractive approach to assessment of stress in animals (43). Results from studies of hair cortisol content of cattle have shown significant elevations when stocking density was markedly changed (47) but not when the change was minor (48) and similarly inconsistent findings have been reported for castration of calves [e.g., (49, 50)]. It seems that when there is a major source of stress, e.g., heat and water deprivation in sheep (51), there is an elevation in hair cortisol content and, likewise, hair cortisol content was associated with clinical disease and pregnancy (52) and with the duration of clinical disease (53) in cows. However, Tracy Burnett and her co-authors (52) pointed out that this parameter did not differentiate lower magnitudes of stress or sub-clinical disease in cattle. Hair cortisol content does show promise as an indicator of animal welfare status but clearly there is a need to develop sampling protocols (such as those suggested by 42) and to be aware of its possible limitations.

### Serotonin

Serotonin, which is actually 5-hydroxytryptramine (5-HT), is derived from tryptophan. It is a neurotransmitter, produced by the serotonergic neurons, but is also formed in a variety of tissues and appears in the circulation from which it is readily removed by platelets or endothelial cells in the lungs and liver. In mammals, there have been numerous studies relating plasma serotonin levels to stress and other disorders. However, as shown in the extensive review of their relation to various pathological states in horses (54), the picture in relation to stress is confusing. Nevertheless, a study of plasma serotonin levels in dairy cows showed the values to be elevated by the stress of negative energy balance (55). This seems to be a measure with potential for detecting the existence of stress conditions in farm animals although the involvement of platelets in metabolism of serotonin means that measurement of free serotonin concentrations is best done with platelet-poor plasma.

### Cardiac Function

The acute response to stressors is an elevation of activity of the sympathetic-adrenal medulla (SAM) axis, most readily detected as an increase in heart rate. Heart rate is easy to measure with electronic recording devices and can be stored on data loggers attached to the animal or transmitted to distant recorders. Heart rate *per se* probably does not provide useful information about long-term welfare status but there is interest in heart rate variation (HRV) as a measure of welfare. HRV is simply obtained by a Fourier transformation of data from any continuous (preferably at least 5 min) heart rate recording. It is alleged that HRV provides information about the balance of activity between the two divisions of the autonomic nervous system: sympathetic and parasympathetic. Or, simply, the balance between sympathetic and vagal activity. Use of HRV for assessment of welfare of farm animals has been comprehensively reviewed (56) and the evidence obtained from about a decade of investigation provided a strong case for continued development of this technology for use in farm animals.

### Infra-Red Thermography (IRT)

Infra-red thermography (IRT) is based on photography of the external surfaces of animals using an infra-red camera. The thermal image can be reproduced in color to reveal the surface heat transfer and blood flow. Initial users of the technique were able to obtain early detection of clinical disease (57). The technique can be used on any region of the body surface, however the eye and surrounding skin tissue provide an image that may reflect the sympathetic-vagal balance of the animal (58). In general, disturbances in thermal radiation from the various surfaces of animals indicate the presence of inflammatory processes, although the genitalia may provide indicators of reproductive status. An overview of use of IRT in farm animals has been provided by (59). Although it is non-invasive and simple to perform, IRT currently shows most promise mainly as a tool for early detection of disease. One of the problems encountered with the technique is standardizing the positioning (angle and distance) of the camera whilst minimizing the need for restraint of the animals.

A variant of IRT is use of functional near infra-red spectroscopy (fNIRS) probes to determine differences in oxy-hemoglobin and deoxy-hemoglobin between right and left cerebral hemispheres of the sheep brain. This has been used to detect a bilateral increase in cerebral activity in response to anticipation of a food reward together with a greater haemodynamic response in the right hemisphere compared with the left (23).

### Other Measures of Sympathetic-Vagal Activity

In addition to IRT other non-invasive measures of sympathetic-vagal activity include proportion of eye white and eye temperature. Percentage of visible eye white in cows increased with increasing frustration (60) however, Gómez et al. (61) found no relation between this measure or eye temperature of cows experiencing either non-stressful (feeding) or stressful (claw trimming) experiences. Likewise, there were no emotion-related effects on percentage of eye white in sheep (62), although the latter authors suggested that eye aperture (possibly related to eyelid muscle tension) may be meaningful (24). Another measure that comes under this heading is body surface humidity which also seems to vary, particularly in concert with the level of sympathetic activity (24, 62).

### Markers of Immune Function

As well as the obvious involvement of immunological mechanisms in response to the presence of pathogenic antigens, the immune system has important functional links with brain function [e.g., emotional limbic system activity (63)] and with the HPA axis (64), the latter particularly through the immunosuppressive activity of glucocorticoids. Brain function is impacted directly by neuroinflammation arising from associated immune dysregulation (65). Markers of immune function include immunoglobulins (e.g., immunoglobulin-A) and the cytokines. The cytokines have been grouped into eight families: interleukins, tumor necrosis factors, interferons, chemokines, haematopoietins, colony stimulating factors, neurotrophins, and growth factors (66). Immune activation may be considered as a stress response in its own right and because many of the markers mentioned above can be measured in samples of blood or saliva, this has become a potentially rich avenue for monitoring well-being of farm animals.

### Salivary Alpha Amylase

There is much interest in the concept, particularly in the human-related literature, that levels of alpha amylase, a digestive enzyme that is present in saliva, provide a marker of sympathetic nerve activity (67). Among farm animals, this measure of stress has been applied particularly to pigs [e.g., (68)] and a study of sheep (69) showed a significant elevation in concentration of salivary alpha amylase 15 min after exposure to a barking dog stimulus. Nevertheless, both of the studies cited above reported considerable individual variation in the responses and indicated that the measure needs further investigation to confirm its reliability as a monitor of stress in farm animals.

### Acute Phase Proteins

Acute phase proteins are a group of approximately 30 mainly liver-derived proteins present in blood that experience a change (25% or more) in concentration in response to inflammation, or specifically in response to altered activity of pro-inflammatory cytokines—particularly interleukin-6, but also interleukin-1, tissue necrosis factor alpha and interferon gamma. Members of this group of proteins include C-reactive protein, serum amyloid A and haptoglobin. Their functions include: enabling entrapment of microorganisms and their products, activation of the complement system, binding cellular remnants, neutralizing enzymes, scavenging free hemoglobin and radicals, and modulation of the immune response (70). Specifically, the acute phase proteins are useful indicators of animal ill health and tissue damage, thus providing information about the severity of the condition and on the degree of recovery or healing that is occurring. Such information is likely to be relevant to assessment of the level of stress experienced by an animal.

### Assessment of Pain

It is generally accepted that the definition of pain in humans, i.e., “an unpleasant sensory and emotional experience associated with actual or potential tissue damage, or described in terms of such damage,” has to be applied equally to other animals. The occurrence of pain experienced by animals is clearly a welfare concern that can be managed effectively only if there are sound means to recognize and quantify it. Presently, for both humans and animals, there are no universally accepted methods for achieving this. Management procedures for farm animals that are perceived (by humans) as being painful usually require use of analgesic drugs or local anesthetics to block potential pain. However, the monitoring of grazing animals to determine when they are experiencing pain “in the field” is largely dependent on assessment of their behavior.

#### Facial Expression

Although it is assumed that the apparent ‘stoicism’ of grazing animals may have been acquired to protect injured animals from the attention of prey species, it has not been a complete impediment to behavioral assessment of grazing animals as a means for detecting pain. Automation of behavioral assessment and its application has been briefly discussed above. Analysis of facial expression has been applied successfully with sheep to determine the effectiveness of this approach for animals with footrot or mastitis (71). Likewise a ‘sheep grimace scale' correlated well with the occurrence of post-surgical pain in sheep (72). There has been some solid progress in advancing the automation and sophistication of this technology [see work with sheep by Lu et al., (73)] and the topic has been recently reviewed in the wider context of farm animal welfare by McLennan (74).

#### Electroencephalography

Because pain is a sensory experience, it manifests at the level of the cerebral cortex so that any technology that provides information on brain function at this level could be used to assess the magnitude of pain (75). Currently, non-invasive imaging of the brain based on computed tomography (CT) or magnetic resonance (MRI) does not appear to provide sufficient resolution for this type of assessment. However, neurophysiological techniques do show promise for assessment of pain in animals (76). These include electroencephalography (EEG) and magnetoencephelography (MEG) (76). EEG has proven useful as a tool for monitoring depth of anesthesia to ensure that the patient is unaware. However, it has been applied also to the identification of nociceptive, i.e., painful, stimuli. Considerable variation occurs with data from EEG recording in animals and this has to be countered by use of highly standardized procedures in association with halothane anesthesia (76). These concerns have limited the usefulness of this technology for monitoring pain. However, when used in conjunction with somatosensory evoked potentials that are generated by various stimuli applied to the skin or other peripheral tissues, especially those evoked by lasers, there has been useful progress in understanding of pain pathways and of the processing of painful stimuli in animals (76).

### Domain-Based Assessment of Animal Welfare

A device for quantifying an animal's overall state of welfare is the so-called Five Domains Model (77). This is a systematic scoring of welfare-significant internal states, labeled as Domains 1–3 (e.g., Nutrition, Environment and Health) plus welfare-significant external circumstances (Domain 4–e.g., Behavior). Once these are identified, any associated affective experiences are accumulated into Domain 5 (e.g., Mental State). A nice account of how this approach was used to evaluate adverse effects of husbandry and other interventions in horses has been provided by McGreevy et al. (78). The authors indicated that the model requires some effort to refine the scoring parameters but it can certainly be extended to various species of animals and could provide a more holistic assessment of welfare than previous approaches.

## Conclusions

There is no perfect remedy for providing objective measures of welfare in animals generally and this obviously applies equally to grazing ruminants. It is likely that the methodology for assessing welfare will utilize a variety of tools, rather than being reliant on a single measure. All of the alternative measures or approaches mentioned in this review show promise for this role and are undergoing further refinement and development, but their reliability is currently generally confined to situations where the degree of compromise to welfare is already severe. All is not lost however. In many cases the particular measure can be applied to studies where data from groups of animals can be ranked to provide information about aversions and unfavorable environments or circumstances that reduce animal welfare or, similarly, about preferences or environments that enhance their welfare.

## Author Contributions

The author confirms being the sole contributor of this work and has approved it for publication.

### Conflict of Interest Statement

The author declares that the research was conducted in the absence of any commercial or financial relationships that could be construed as a potential conflict of interest.

## References

[1] Moberg GP A model for assessing the impact of behavioural stress on domestic animals. J Anim Sci. (1987) 65:1228–35. 10.2527/jas1987.6551228x 3693149

[2] Broom DM Johnson KG Stress and Animal Welfare. London: Chapman and Hall (1993).

[3] Broom DM Assessing the welfare of modified or treated animals. Livest Prod Sci. (1993) 36:39–54. 10.1016/0301-6226(93)90136-6

[4] Dawkins MS La Souffrance Animale. Maisons-Alfort: Editions du Point Vétérinaire (1983).

[5] Fraser D Science, values and animal welfare: exploring the inextricable connection. Anim Welfare. (1995) 4:103–17.

[6] Désiré L Boissy A Veissier I. Emotions in farm animals: a new approach to animal welfare in applied ethology. Behav Proc. (2002) 60:165–80. 10.1016/S0376-6357(02)00081-5 12426068

[7] Webb LE Veenhoven R Harfeld JL Jensen MB. What is animal happiness? Ann NY Acad Sci. (2019) 1438:62–78. 10.1111/nyas.13983 30345570 PMC7379717

[8] Safina C Beyond Words: What Animals Think and Feel. New York, NY: Henry Holt & Co (2015).

[9] Dawkins MS Animal Suffering: The Science of Animal Welfare. Dordrecht: Springer Netherlands (1980).

[10] Alvåsen K Doohoo I Roth A Emanuelson U. Farm characteristics and management routines related to cow longevity: a survey among Swedish dairy farmers. Acta Vet Scand. (2018) 60:38. 10.1186/s13028-018-0390-8 29914547 PMC6006783

[11] McGrath J Duval SM Tamassia LFM Kindermann M Stemmler RT de Gouvea VN. Nutritional strategies in ruminants; a lifetime approach. Res Vet Sci. (2018) 116:28–39. 10.1016/j.rvsc.2017.09.011 28943061

[12] Dawkins MS The science of animal suffering. Ethology. (2008) 114:937–45. 10.1111/j.1439-0310.2008.01557.x

[13] Boissy A Manteuffel G Jensen MB Moe RO Spruijt B Keeling LJ. Assessment of positive emotions in animals to improve their welfare. Physiol Behav. (2007) 92:375–97. 10.1016/j.physbeh.2007.02.003 17428510

[14] Ginane C Bonnet M Baumont R Revell K Feeding behaviour in ruminants: a consequence of interactions between a reward system and the regulation of metabolic homeostasis. Anim Prod Sci. (2015) 55:247–60. 10.1071/AN14481

[15] Candiani D Salamano G Mellia E Doglione L Bruno R Toussaint M. A combination of behavioral and physiological indicators for assessing pig welfare on the farm. J Appl Anim Welfare Sci. (2008) 11:1–13. 10.1080/10888700701729080 18444023

[16] Barragan AA Piñeiro JM Schuenemann GM Rajala-Schultz PJ Sanders DE Lakritz J Assessment of daily activity patterns and biomarkers of pain, inflammation, and stress in lactating dairy cows diagnosed with clinical mastitis. J Dairy Sci. (2018) 101:8248–58. 10.3168/jds.2018-14510 29937269

[17] Williams J Ntallaris T Routly JE Jones DN Cameron J Holman-Coates A. Association of production diseases with motor activity-sensing devices and milk progesterone concentrations in dairy cows. Theriogenol. (2018) 118:57–62. 10.1016/j.theriogenology.2018.05.038 29885641

[18] Müller E Münger A Mandel R Eggerschwiler L Schwinn A-C Gross JJ. Physiological and behavioural, responses of dairy cows to an acute metabolic challenge. J Anim Physiol Anim Nutr. (2018) 102:1120–30. 10.1111/jpn.12931 29907969

[19] Meunier B Pradel P Sloth KH Cirié C Delval E Mialon MM Image analysis to refine measurements of dairy cow behaviour from a real-time location system. Biosyst Eng. (2018) 173:32–44. 10.1016/j.biosystemseng.2017.08.019

[20] Manteuffel G Puppe B Schön PC Vocalization of farm animals as a measure of welfare. Appl Anim Behav Sci. (2004) 88:163–82. 10.1016/j.applanim.2004.02.012

[21] Briefer EF Vocal expressions of emotions in mammals: mechanisms of production and evidence. J Zool. (2012) 288:1–20. 10.1111/j.1469-7998.2012.00920.x

[22] Green AC Johnston IN Clark CEF. The evolution of cattle bioacoustics and application for advanced dairy systems. Animal. (2018) 12:1250–9. 10.1017/S1751731117002646 29065943

[23] Chincarini M Qiu L Spinelli L Torricelli A Minero M Dalla Costa E. Evaluation of sheep anticipatory response to a food reward by means of functional near-infrared spectroscopy. Animals. (2019) 9:11. 10.3390/ani9010011 30597931 PMC6356716

[24] Reefmann N Wechsler B Gygax L Behavioural and physiological assessment of positive and negative emotion in sheep. Physiol Behav. (2009) 98:651–8. 10.1016/j.anbehav.2009.06.015 19490924

[25] Phythian CJ Michalopoulou E Cripps PJ Duncan JS Wemelsfelder F On-farm qualitative behavipour assessment in sheep: repeated measurements across time, and association with physical indicators of flock health and welfare. Appl Anim Behav Sci. (2016) 175:23–31. 10.1016/j.applanim.2015.11.013

[26] Collins T Stockman CA Barnes AL Miller DW Wickham SL Fleming PA. Qualitative behavioural assessment as a method to identify potential stressors during commercial sheep transport. Animals. (2018) 8:209. 10.3390/ani8110209 30445737 PMC6262568

[27] Clegg ILK. Cognitive bias in zoo animals: an optimistic outlook for welfare assessment. Animals. (2018) 8:104. 10.3390/ani8070104 29954151 PMC6071086

[28] McBride SD Morton AJ Visual attention and cognitive performance in sheep. Appl Anim Behav Sci. (2018) 206:52–8. 10.1016/j.applanim.2018.05.026

[29] Monk JE Belson S Colditz IG Lee C. Attention bias test differentiates anxiety and depression in sheep. Front Behav Neurosci. (2018) 12:246. 10.3389/fnbeh.2018.00246 30405371 PMC6205987

[30] Monk JE Lee C Belson S Colditz IG Campbell DLM. The influence of pharmacologically-induced affective states on attention bias in sheep. PeerJ. (2019) 7:e7033. 10.7717/peerj.7033 31211015 PMC6557257

[31] Raoult CMC Gygax L. Mood induction alters attention toward negative-positive stimulus pairs in sheep. Sci Rep. (2019) 9:7759. 10.1038/s41598-019-44330-z 31123314 PMC6533262

[32] Lecorps B Ludwig BR von Keyserlingk MAG Weary DM Pain-induced pessimism and anhedonia: evidence from a novel probability-based judgement bias test. Front Behav Neurosci. (2019) 13:54 10.3389/fnbeh.2019.00054 30949035 PMC6435490

[33] Bali A Randhawa PK Jaggi AS. Stress and opioids: role of opioids in modulating stress-related behavior and effect of stress on morphine conditioned place preference. Neurosci Biobehav Rev. (2015) 51:138–50. 10.1016/j.neubiorev.2014.12.018 25636946

[34] Rotondo F Butz H Syro LV Yousef GM Di Ieva A Restrepo LM. Arginine vasopressin (AVP): a review of its historical perspectives, current research and multifunctional role in the hypothalamo-hypophysial system. Pituitary. (2016) 19:345–55. 10.1007/s11102-015-0703-0 26762848

[35] Peckett AJ Wright DC Riddell MC The effects of glucocorticoids on adipose tissue lipid metabolism. Metabolism. (2011) 80:1800–10. 10.1016/j.metabol.2011.06.012 21864867

[36] Stafford KJ Mellor DJ. The welfare significance of the castration of cattle: a review. NZ Vet J. (2005) 53:271–8. 10.1080/00480169.2005.36560 16220117

[37] Mellor DJ Murray L. Effects of tail docking and castration on behaviour and plasma cortisol concentrations in young lambs. Res Vet Sci. (1989) 46:387–91. 10.1016/S0034-5288(18)31185-8 2544971

[38] Ralph CR Tilbrook AJ. The usefulness of measuring glucocorticoids for assessing animal welfare. J Anim Sci. (2016) 94:457–70. 10.2527/jas.2015-9645 27065116

[39] Ingram JR Matthews LR McDonald RM Remote blood sampling device: a stress free blood sampling technique for free ranging animals. Proc N Z Soc Anim Prod. (1994) 54:39–42.

[40] Van Reenen CG O'Connell NE Van der Werf JTN Korte SM Hopster H Jones RB. Responses of calves to acute stress: individual consistency and relations between behavioral and physiological measures. Physiol Behav. (2005) 85:557–70. 10.1016/j.physbeh.2005.06.015 16081113

[41] Morrow CJ Kolver ES Verkerk GA Matthews LR. Fecal glucocorticoid metabolites as a measure of adrenal activity in dairy cattle. Gen Comp Endocrinol. (2002) 126:229–41. 10.1006/gcen.2002.7797 12030779

[42] Palme R Robia C Baumgartner W Mostl E. Transport stress in cattle as reflected by an increase in faecal cortisol metabolite concentrations. Vet Rec. (2000) 146:108–9. 10.1136/vr.146.4.108 10682697

[43] Heimbürge S Kanitz E Otten W. The use of hair cortisol for the assessment of stress in animals. Gen Comp Endocrinol. (2019) 270:10–7. 10.1016/j.ygcen.2018.09.016 30287191

[44] Slominski A Wortsman J Tuckey RC Paus R. Differential expression of HPA axis homolog in the skin. Mol Cell Endocrinol. (2007) 265:143–9. 10.1016/j.mce.2006.12.012 17197073 PMC1839836

[45] Meyer JS Novak MA. Hair cortisol: a novel biomarker of hypothalamic-pituitary-adrenocortical activity. Endocrinol. (2012) 153:4120–7. 10.1210/en.2012-1226 22778226 PMC3423616

[46] Harkey MR. Anatomy and physiology of hair. Forens Sci Int. (1993) 63:9–18. 10.1016/0379-0738(93)90255-9 8138238

[47] Schubach KM Cooke RF Brandäo AP Lippolis KD Silva LGT Marques RS. Impacts of stocking density on development and puberty attainment of replacement beef heifers. Animal. (2017) 11:2260–7. 10.1017/S1751731117001070 28521848

[48] Silva PRB Lobeck-Luchterhand KM Cerri RLA Haines DM Ballou MA Endres MI Effects of prepartum stocking density on innate and adaptive leucocyte responses and serum and hair cortisol concentrations. Vet Immunol Immunopath. (2016) 169:39–46. 10.1016/j.vetimm.2015.11.007 26827837

[49] Creutzinger KC Stookey JM Marfleet TW Campbell JR Janz DM Marques FI An investigation of hair cortisol as a measure of long term stress in beef cattle: results from a castration study. Can J Anim Sci. (2017) 97:499–509. 10.1139/CJAS-2016-0206

[50] Marti S Meléndez DM Pajor EA Moya D Heuston CEM Gellatly D Effect of band and knife castration of beef calves on welfare indicators of pain at three relevant industry ages: II. Chronic pain. J Anim Sci. (2017) 96:4367–80. 10.2527/jas.2017.1763 29108039

[51] Ghassemi-Nejad J Lohakare JD Son JK Kwon EG West JW Sung KI. Wool cortisol is a better indicator of stress than blood cortisol in ewes exposed to heat stress and water restriction. Animal. (2014) 8:128–32. 10.1017/S1751731113001870 24182313

[52] Burnett TA Madureira AML Silper BF Tahmasbi A Nadalin A Veira DM. Relationship of concentrations of cortisol in hair with health, biomarkers in blood, and reproductive status in dairy cows. J Dairy Sci. (2015) 98:4414–26. 10.3168/jds.2014-8871 25958283

[53] Braun U Clavadetscher G Baumgarter MR Riond B Binz TM. Hair cortisol concentration and adrenal gland weight in healthy and ill cows. Schw Archiv Tierheil. (2017) 159:493–5. 10.17236/sat00128 28952959

[54] Ambrojo KS Poggi JCG Corzano MM Clinical Aspects Related to Plasma Serotonin in the Horse. London: IntechOpen (2018).

[55] Bruschetta G Di Pietro P Sanzarello L Giacoppo E Ferlazzo AM Plasma serotonin levels in Italian Friesian dairy cows. Vet Res Comm. (2010) 34(Suppl. 1):S17–20. 10.1007/s11259-010-9371-8 20449652

[56] Von Borell E Langbein J Després G Hansen S Leterrier C Marchant-Forde J. Heart rate variability as a measure of autonomic regulation of cardiac activity for assessing stress and welfare in farm animals - a review. Physiol Behav. (2007) 92:293–316. 10.1016/j.physbeh.2007.01.007 17320122

[57] Schaefer AL Cook NJ Tessaro SV Deregt D Desroaches G Dubeski P Early detection and prediction of infection using infrared thermography. Can J Anim Sci. (2004) 84:73–80. 10.4141/A02-104

[58] Stewart M Webster JR Verkerk GA Schaefer AL Colyn JL Stafford KJ. Non-invasive measurement of stress in dairy cows using infrared thermography. Physiol Behav. (2007) 92:520–5. 10.1016/j.physbeh.2007.04.034 17555778

[59] McManus C Tanure CB Peripolli V Seixas L Fischer V Gabbi AM Infrared thermography in animal production: a review. Comput Electron Agric. (2016) 123:10–6. 10.1016/j.compag.2016.01.027

[60] Sandem AL Braastad BO Bue KE Eye white may indicate emotional state on a frustration-contentedness axis in dairy cows. Appl Anim Behav Sci. (2002) 79:1–10. 10.1016/S0168-1591(02)00029-1

[61] Gómez YB Vieler R Hankele AK Zähner M Savary P Hillmann E Evaluation of visible eye white and maximum eye temperature as non-invasive measures of stress in dairy cows. Appl Anim Behav Sci. (2018) 198:1–8. 10.1016/j.applanim.2017.10.001

[62] Reefmann N Bütikofer Kaszas F Wechsler B Gygax L Physiological expression of emotional reactions in sheep. Anim Behav. (2009) 78:235–41. 10.1016/j.physbeh.2009.05.017 19490924

[63] Haas HS Schauenstein K. Neuroimmunomodulation via limbic structures – the neuroanatomy of psychoimmunology. Progr Neurobiol. (1997) 51:195–222. 10.1016/S0301-0082(96)00055-X 9247964

[64] Bellavance MA Rivest S. The HPA-immune axis and the immunomodulatory actions of glucocorticoids in the brain. Front Immunol. (2014) 5:136. 10.3389/fimmu.2014.00136 24744759 PMC3978367

[65] Ramsohoff RM Schafer D Vincent A Blachère NE Bar-or A Neuroinflammation: ways in which the immune system affects the brain. Neurotherap. (2015) 12:896–909. 10.1007/s13311-015-0385-3 PMC4604183 26306439

[66] Turnbull AV Rivier CL Regulation of the hypothalamic-pituitary-adrenal axis by cytokines: actions and mechanism of action. Physiol Rev. (1999) 79:1–71. 10.1152/physrev.1999.79.1.1 9922367

[67] Rohleder N Nater UM Wolf JM Ehlert U Kirschbaum C. Psychosocial stress-induced activation of salivary alpha-amylase: an indicator of sympathetic activity? Ann NY Acad Sci. (2004) 1032:258–63. 10.1196/annals.1314.033 15677423

[68] Contreras-Aguilar MD Escribano D Martinez-Subiela S Martinez-Miró S Cerón JJ Tecles F. Changes in alpha-amylase activity, concentration and isoforms in pigs after an experimental acute stress model: an exploratory study. BMC Vet Res. (2018) 14:256. 10.1186/s12917-018-1581-2 30157843 PMC6116453

[69] Fuentes-Rubio M Fuentes F Otal J Quiles A Hevia ML Validation of an assay for quantification of alpha-amylase activity in saliva of sheep. Can J Vet Res. (2016) 80:197–202. 27408332 PMC4924553

[70] Jain S Gautam V Naseem N. Acute-phase proteins: as diagnostic tool. J Pharm Bioallied Sci. (2011) 3:118–27. 10.4103/0975-7406.76489 21430962 PMC3053509

[71] Mclennan KM Rebelo CJB Corke MJ Holmes MA Leach MC Constantino-Casa F Development of facial expression scale using footrot and mastitis as models of pain in sheep. Appl Anim Behav Sci. (2016) 176:19–26. 10.1016/j.applanim.2016.01.007

[72] Häger C Biernot S Buettner M Glage S Keubler LM Held N. The sheep grimace scale as an indicator of post-operative distress and pain in laboratory sheep. PLoS ONE. (2017) 12:e0175899. 10.1371/journal.pone.0175839 28422994 PMC5396914

[73] Lu Y Mahmoud M Robinson P Estimating sheep pain level using facial action unit detection. In: 12th International Conference on Automatic Face and Gesture Recognition. Washington, DC (2017), p. 394–99. 10.1109/FG.2017.56

[74] McLennan KM Why pain is still a welfare issue for farm animals, and how facial expression could be the answer. Agriculture. (2018) 8:127–45. 10.3390/agriculture8080127

[75] Schnitzler A Ploner M. Neurophysiology and functional neuroanatomy of pain perception. J Clin Neurophys. (2000) 17:592–603. 10.1097/00004691-200011000-00005 11151977

[76] Murrell JC Johnson CB. Neurophysiological techniques to assess pain in animals. J Vet Pharm Therap. (2006) 29:325–35. 10.1111/j.1365-2885.2006.00758.x 16958776

[77] Mellor DJ Operational details of the Five Domains Model and its key applications to the assessment and management of animal stress. Animals. (2017) 7:60 10.3390/ani7080060 PMC5575572 28792485

[78] McGreevy P Berger J de Brauwere N Doherty O Harrison A Fiedler J. Using the Five Domains Model to assess the adverse impacts of husbandry, veterinary, and equitation interventions on horse welfare. Animals. (2018) 8:41. 10.3390/ani8030041 29562654 PMC5867529

